# Noninvasive Strategies to Optimise Brain Plasticity: From Basic Research to Clinical Perspectives

**DOI:** 10.1155/2013/863970

**Published:** 2013-12-30

**Authors:** Alessandro Sale, Anthony J. Hannan, Lamberto Maffei, Andrea Guzzetta

**Affiliations:** ^1^Neuroscience Institute, National Research Council (CNR), 56124 Pisa, Italy; ^2^Florey Institute of Neuroscience and Mental Health, Melbourne Brain Centre, University of Melbourne, Parkville, VIC 3010, Australia; ^3^SMILE Lab, Department of Developmental Neuroscience, IRCCS Stella Maris Scientific Institute, 56128 Calambrone (Pisa), Italy

Brain plasticity can be defined as the capacity of cerebral neurons and neural circuits to change, structurally and functionally, in response to experience. This fundamental property is essential for maturation of sensory functions during development, for the adaptability of our behaviour to the environment through learning and memory processes and for brain repair in response to disease and trauma.

Given its relevance for primary brain processes, it is not surprising that great effort is being made in multiple laboratories to elaborate intervention procedures aimed at enhancing neural plasticity in the brain. In addition to their theoretical relevance, these studies may pave the way for novel paradigms or therapeutic agents for rehabilitation and recovery from nervous system injury. Among the possible experimental approaches that can be used to promote brain plasticity, of great relevance are those based on noninvasive procedures characterised by their capability to boost the potential for plasticity retained by neural circuitries without being associated with dangerous side effects. Some paradigms appear particularly worthy of interest, in light of their powerful impact on brain health, and include exposure to enriched environments characterised by high levels of sensory, motor, and cognitive stimulation, behavioural interventions based on the enhancement of sensory stimuli (such as perceptual learning), and dietary manipulations aimed at the optimisation of caloric intake and food balance.

One of the most exciting findings resulting from the application of these procedures is the demonstration, obtained primarily in the paradigmatic visual system but extending to other systems and functions, that the adult brain is not “hard-wired” with immutable neuronal circuits but can be pushed to unfold a high degree of plasticity even well past the end of the so-called “critical periods,” sensitive phases during early development when plasticity levels are particularly high. This special issue provides a collection of several papers addressing the impact of a number of noninvasive procedures on developmental and adult brain plasticity, with a focus on both animal models and human research.

J. Bonaccorsi et al. concentrated on system consolidation, a crucial mechanism mediated by the hippocampus and other medial temporal lobe structures and underlying the precise recall of already acquired memories. The authors contributed a very innovative research paper providing the first evidence that exposure of adult mice to environmental enrichment affects the time-dependent process of spatial memory system consolidation, inducing an earlier recruitment of the medial prefrontal cortex and also the progressive activation of a distributed cortical network that is not activated in mice reared in standard housing conditions.

Antidepressant drugs such as the selective serotonin reuptake inhibitor (SSRI) fluoxetine (Prozac) have been recently shown to have a major impact on brain plasticity, qualifying as powerful enviromimetics, substances that can be used to mimic the beneficial effects induced by exposure to environmental enrichment. In particular, a chronic treatment with fluoxetine has been previously shown to reopen forms of juvenile-like plasticity in the adult visual cortex of rodents. In this special issue, E. Tiraboschi et al. continued in this established research field by using fluoxetine-induced plasticity in the adult rat visual cortex as an experimental model to investigate possible modulatory effects on gene expression by means of microarrays and RT-PCR. They provide evidence that the combination of fluoxetine and monocular deprivation (i.e., the closure of one eye) induces significant changes in the expression of genes belonging to different biological classes, such as chromatin structure remodelling, transcription factors, extracellular matrix, and excitatory and inhibitory neurotransmission.

Closely related to the paper by E. Tiraboschi et al., J. F. Maya-Vetencourt provides a review article on the emerging role in brain plasticity played by the recently discovered neuronal-specific and activity-dependent transcription factor NPAS4, which has been proved to be involved in as various processes as neural circuits' reorganization after cerebral ischemia and brain injury, amygdala and hippocampal-dependent memory, homeostatic plasticity, and neurogenesis. The author provides a useful discussion of the link between NPAS4 expression and the regulation of GABA-mediated inhibitory transmission and discusses how future lines of research might concentrate on NPAS4 as a possible mediator for the established effects of environmental enrichment and fluoxetine administration on adult visual cortex plasticity.

When looking at the effects of environmental and pharmacological manipulations, one critical factor to be considered is the possibility that the selected intervention procedures might also induce unpredictable amounts of undesired stress, which can neutralise their impact on brain and behaviour. While the literature on the impact of acute and chronic stress is huge, one major challenge is to understand the conditions under which the harmful effects of a stressful situation can be converted into a potential benefit for brain plasticity. S. Capoccia et al. provide a research paper investigating the effects of stressors of different nature and length on hippocampal plasticity, with the associated changes in the immune and neuroendocrine activation. The authors demonstrate that while prolonged stress in mice is associated with immunosuppression and lowering of brain-derived neurotrophic factor (BDNF) levels, opposite changes are elicited by brief exposure to stressful stimuli. The results are discussed in terms of possible hormetic effects set in motion by mild stress, resulting in the activation of a greater flexibility for resource management in moderately challenging environmental conditions.

One fundamental source of modification of the extracellular and intracellular milieu is food intake and the ensuing modulation of energy metabolism. Dietary factors are increasingly recognised as powerful regulators of neural plasticity, exerting their effects on the brain by affecting molecular events related to synaptic plasticity, neuronal signalling, and, ultimately, mental health. M. Mainardi et al. contribute with a timely review on this exciting matter, focusing on the literature dealing with neural plasticity induced by environmental stimulation (e.g., environmental enrichment, physical exercise, dietary restriction, and high-fat diet) on the arcuate nucleus of the hypothalamus, the primary sensor of plasmatic leptin levels.

The special issue also contains significant contributions centred on the effects of environment on brain plasticity in humans. E. Inguaggiato et al. surveyed the literature on the impact of noninvasive rehabilitation strategies in children with unilateral cerebral palsy, providing the first review on this subject. The selected literature discussed by the authors employed totally nonpharmacological procedures such as constraint-induced movement therapy, occupational therapy, motor training, and virtual reality exposure.

One of the most common and severely disabling neural diseases is stroke, a leading cause of permanent adult disability. Enhancing neural plasticity in patients with stroke might considerably affect their functional output, boosting the recovery process by eliciting and facilitating the spontaneous reparative potential of the brain. In their review, F. Faralli et al. discuss how, and to what extent, noninvasive intervention strategies such as mirror therapy, action observation, and mental practice affect poststroke recovery. The review nicely integrates preclinical studies with clinical evidence, bridging the translational gap and providing a list of possible molecular factors underlying the beneficial effects elicited by environmental stimulation.

An emerging and very attractive area of research in experience-dependent neuroplasticity is meditation, which appears to elicit plasticity processes affecting higher cognitive functions. The research paper by M. R. Hagerty et al. reports an fMRI and EEG study of the brain of a trained meditator in the course of ecstatic meditation during a Buddhist concentration technique called jhana. The authors document the areas activated during this practice and relate them to the subjective reports of emotions and psychophysical states. A striking finding is the activation of the dopamine/opioid reward system during meditation stages corresponding to subjective reports of joy, particularly relevant if one considers that it is achieved through a totally self-stimulating procedure based on internal mental processes.

Our hope is that this special issue will serve to emphasize the relevance of environment-based intervention strategies in eliciting brain plasticity under both physiological and pathological conditions. This area of investigation will likely emerge as one of the most successful in the fields of brain repair, neurology, psychiatry, and mental health.

*Alessandro Sale* *Alessandro Sale* *Anthony J. Hannan* *Anthony J. Hannan* *Lamberto Maffei* *Lamberto Maffei* *Andrea Guzzetta* *Andrea Guzzetta*

